# Interleukin−1 receptor antagonist and heat shock protein 90alfa are independently associated with fatigue and general health in euthyroid Hashimoto’s thyroiditis

**DOI:** 10.3389/fendo.2026.1862441

**Published:** 2026-07-02

**Authors:** Roald Omdal, Tore Grimstad, Grete Jonsson, Jan Terje Kvaløy, Charlotte Gibbs, Laurens C. Reitsma, Tomm Bernklev, Geir Hoff

**Affiliations:** 1 Research Department, Stavanger University Hospital, Stavanger, Norway; 2 Department of Clinical Science, University of Bergen, Bergen, Norway; 3 Department of Internal Medicine, Stavanger University Hospital, Stavanger, Norway; 4 Department of Medical Biochemistry, Stavanger University Hospital, Stavanger, Norway; 5 Department of Mathematics and Physics, University of Stavanger, Stavanger, Norway; 6 Department of Medicine, Østfold Hospital Trust, Kalnes, Norway; 7 Department of Surgery, Akershus University Hospital, Lørenskog, Norway; 8 Institute of Clinical Medicine, University of Oslo, Oslo, Norway; 9 Department of Research and Innovation, Telemark Hospital, Skien, Norway

**Keywords:** autoimmune thyroid disease, biomarkers, fatigue, Hashimoto’s thyroiditis, heat shock protein 90α (HSP90α), IL-1Ra, interleukin-1, patient-reported outcomes

## Abstract

**Background:**

Patients with Hashimoto’s thyroiditis frequently experience fatigue and impaired health-related quality of life (HRQOL) despite biochemical euthyroidism, suggesting that immune-mediated mechanisms beyond thyroid hormone deficiency contribute to symptom generation. The role of specific inflammatory mediators - including interleukin-1 receptor antagonist (IL-1Ra), heat shock protein 90α (HSP90α), interleukin-6 (IL-6), tumor necrosis factor-α (TNF-α), and anti-TPO antibodies - in relation to patient-reported outcomes (PROMs) has not been systematically examined.

**Methods:**

In a cross-sectional study of 36 euthyroid patients with confirmed Hashimoto’s thyroiditis, serum/plasma levels of IL-1Ra, HSP90α, IL-6, and TNF-α were measured alongside anti-TPO. PROMs included the SF-36 General Health subscale (inverted; iGH), the Fatigue Visual Analog Scale (fVAS), and the Fatigue Severity Scale (FSS). Spearman correlations, univariable and multivariable linear regression, and principal component analysis (PCA) were applied.

**Results:**

The cohort displayed severe fatigue at baseline (median fVAS 85, FSS 6.7, SF-36 GH 16). IL-1Ra was the most consistent biomarker correlate of symptom burden, independently associated with poorer general health and more fatigue across all regression models. HSP90α showed an inverse and independent association with fatigue in multivariable models, suggesting a potentially protective role. IL-6 contributed in correlation analysis only, while TNF-α and anti-TPO were not significantly associated with any PROM in any analytical approach.

**Conclusion:**

IL-1Ra and HSP90α, but not anti-TPO, are independently associated with fatigue and general health in euthyroid Hashimoto’s thyroiditis, pointing to IL-1 pathway activity as associated with symptom burden and potentially playing a key role in this condition.

## Introduction

Hashimoto’s thyroiditis is an organ−specific autoimmune thyroid disease characterized by lymphocytic infiltration of the thyroid gland, autoantibodies against thyroid peroxidase (TPO) and thyroglobulin (Tg), and progressive destruction of thyroid follicles. It is the predominant cause of hypothyroidism in iodine−sufficient populations. The global prevalence of Hashimoto’s thyroiditis varies widely, with estimates ranging from approximately 5–10% overall, and reports from individual regions as high as >20% and as low as <0.5%, consistent with a marked female predominance (1). Anti-TPO antibodies (anti-TPO) are considered a sensitive marker of autoimmune thyroid disease and are recommended as a key laboratory test in the diagnosis (2).

Anti-TPO serves not only as a diagnostic marker but may also convey information about disease activity and symptom burden. Anti-TPO has been shown to mediate direct thyrocyte damage through complement-dependent cytotoxicity (CDC) and antibody-dependent cell-mediated cytotoxicity (ADCC), and titer values reflect the degree of intrathyroidal lymphocytic infiltration (3). In addition, anti-TPO antibodies promote pro-inflammatory cytokine production (tumor necrosis factor-alfa (TNF-α), interferon (IFN)-γ) from mononuclear cells and induce oxidative stress, mechanisms that may exert systemic effects beyond the thyroid gland (4). Several studies report that elevated anti-TPO titers are associated with increased symptom burden and reduced HRQOL in euthyroid patients, independent of thyroid function. Ott et al. found that anti-TPO > 121 IU/mL was significantly associated with chronic fatigue, and lower quality of life despite comparable TSH values (5), while another study demonstrated positive correlations between anti-TPO levels and fatigue severity and negative correlations with general health and vitality (6). Müssig et al. reported that anti-TPO positivity predicted poorer psychosocial quality of life over three years (7). In a systematic review 21 out of 30 well-designed studies reported a probable relation between thyroid autoimmunity, and persisting symptoms or lower HRQOL (8). However, it is unclear exactly how anti-TPO exerts this effect, and it is possible that anti-TPO is a surrogate marker for a complex immunological process in which downstream mediators - such as interleukin (IL)-1β, IL-6, and TNF-α - may be more directly relevant to symptom generation.

Beyond biochemical hypothyroidism, patients with Hashimoto’s thyroiditis often experience significant fatigue, cognitive complaints, mood changes, and reduced health-related quality of life (HRQOL). Several studies demonstrate that symptom load and HRQOL remain impaired in a substantial subset of patients despite normalization of thyroid hormone levels on levothyroxine, and even in euthyroid individuals with thyroid autoimmunity (8–11). Consistent with this, our previous study of women undergoing thyroidectomy for benign goiter with underlying Hashimoto’s thyroiditis showed that removal of the thyroid gland led to a marked and sustained improvement in fatigue and HRQOL, largely abolishing the excess fatigue burden (12). Taken together, these observations highlight a dissociation between thyroid function tests and symptoms and strongly suggest that immune−mediated mechanisms, in addition to hormone deficiency, contribute to symptom generation in Hashimoto’s thyroiditis.

Immunopathologically, Hashimoto’s thyroiditis is associated with a Th1− and Th17 response, increased IFN−γ, TNF−α, IL−2, IL−17, and a disrupted regulatory T-cells (Treg) balance, with inflammasome activation and cytokine−mediated tissue damage (13). In other chronic inflammatory states, such mediators drive “sickness behavior” – an evolutionary conserved cerebral response comprising fatigue, anhedonia, reduced activity, and cognitive slowing. It is mediated through effects on cytokine receptors, neuroimmune mechanisms, and neuroendocrine circuits. IL−6 and TNF−α have been linked to fatigue and HRQOL across cardiovascular, pulmonary, and oncologic populations (14, 15), while IL−1 pathway activity- as reflected by IL−1Ra levels - emerges as a particularly consistent correlate of fatigue and depression in some chronic inflammatory and autoimmune conditions (16–18).

​Heat−shock protein 90-alfa (HSP90α) is a ubiquitously expressed chaperone that supports stability and function of many signaling proteins involved in immunity and inflammation, including NF−κB and JAK/STAT pathway components. It exerts proinflammatory effects by promoting production of cytokines such as TNF−α, IL−6 and IL−1β, although its chaperone function can also confer cytoprotective and homeostatic roles in certain contexts (19, 20).

However, little is known about how IL−1Ra, HSP90α, IL−6, and TNF−α relate to fatigue and HRQOL in Hashimoto’s thyroiditis (21). This study addresses this gap by analyzing these biomarkers including anti-TPO in relation to PROMs in a cohort of patients with Hashimoto’s thyroiditis a state characterized by active autoimmune inflammation. Using correlation, regression, and principal component analysis (PCA), the study aims to identify which inflammatory markers are most closely linked to symptom burden, how they interact statistically and potential network patterns.

## Patients and methods

### Study design and cohort

This was a single-center, cross-sectional substudy of baseline data from adults with Hashimoto’s thyroiditis. In the main study, patients were referred to Telemark Hospital by their general practitioner for evaluation regarding inclusion in a study examining the effects of thyroidectomy on general health outcomes (12). The present substudy was restricted to baseline (preoperative) data obtained from blood samples and PROM questionnaires. All 36 patients included were euthyroid at baseline. Nineteen were receiving levothyroxine therapy and 17 were not.

Inclusion criteria were age ≥18 years, serological evidence of thyroid autoimmunity anti-TPO >100 IU/mL (normal, ≤100 IU/mL) and thyroid ultrasound findings compatible with chronic autoimmune thyroiditis.

Exclusion criteria were age <18 years, pregnancy, and being unable to comprehend information adequately to give informed consent.

A total of 36 patients fulfilled these criteria and formed the analysis population.

All participants were given oral and written information about the study and provided written informed consent.

### Patient−reported outcome measures

Three widely used and internationally accepted generic and uni-dimensional fatigue instruments, all with established internal consistency and reliability were used:

SF−36 General Health (GH) subscale. Higher scores denote better perceived health, with a score range from 0-100. For multivariable analyses and PCA, inverted GH scores (iGH calculated as 100 minus GH score) were used so that higher values represent poorer general health, aligning directionally with fatigue measures (fVAS and FSS) (22).

Fatigue Visual analog scale (fVAS) consists of a 100 mm horizontal line with vertical anchors, where the left end (0 mm) is labeled “no fatigue”, and the right end (100 mm) is labeled “fatigue as bad as it can be”. Responders are asked to grade fatigue experienced during the past one week by putting a mark on the line, and the recorded distance in millimeters is the fatigue score (23).

Fatigue Severity Scale (FSS). The FSS consists of nine statements regarding fatigue and are rated on a 7-point Likert scale. The FSS score is the mean score of these nine questions, and ranges from 1 to 7. Higher scores indicate more fatigue (24).

### Biomarker assessment

Venous blood samples for analysis of IL-1Ra and HSP90α were collected in serum tubes and allowed to clot in room temperature for 30–60 minutes before centrifugation (2500G, 10 min, 4 °C). Blood-plasma for analysis of TNFα and IL-6 was collected in EDTA-tubes, kept cold and centrifuged (2500G, 10 min, 4 °C) within 60 minutes. Serum and plasma samples were aliquoted and immediately stored at -20 °C until analysis.

Serum IL-1Ra, plasma TNFα and plasma IL-6 were measured in freshly thawed aliquots by electrochemiluminiscence on Meso^®^ QuickPlex SQ 120 Imager (Meso Scale Diagnostics, Rockville, MD, USA) using the following assay-kits: V-PLEX^®^ Human IL-1RA and S-PLEX^®^ Custom Human Biomarkers TNFα and IL-6 (Meso Scale Diagnostics, Rockville, MD, USA). Serum HSP90α was measured by a commercially available enzyme-linked immunosorbent assay (ELISA, Enzo Life Sciences, Farmingdale, NY, USA). Anti-TPO was measured in the routine laboratory at Telemark Hospital.

An independent in-house control sample was analyzed to assess the different assay’s coefficient of variation (CV). The intra- (n=3-6) and inter-assay CV (n=3) for IL-1Ra was <10%, for HSP90α <5% and < 15%, for TNFα <6% and <7.5%, and for IL-6 <7% and <12% respectively.

### Statistical analyses

Spearman rank correlations were calculated between each PROM (iGH, fVAS, FSS) and each biomarker (anti-TPO, IL−1Ra, IL−6, TNF−α, HSP90α), to describe bivariate relationships and to inform model building. For each PROM, separate linear regression models were fitted with the PROM as response variable and each biomarker as the main predictor, adjusting for age and sex. This yielded age− and sex−adjusted β−coefficients and p−values for the effect of each biomarker considered one at a time. In addition, R^2^ values for each of the biomarker models were calculated, representing the magnitude of explained variance provided by the model. One extreme TNF−α outlier with a biologically implausible value of 5809 was excluded from the regression analyses. Such a value is typically only observed in the context of severe systemic infections or other critical inflammatory conditions. Further, for each PROM a multivariable model including anti-TPO, IL−1Ra, IL−6, HSP90α, TNF−α, age, and sex was fitted. Backward selection was subsequently applied, with age and sex kept in the model as adjustment variables, to derive a model retaining only variables with meaningful contributions. Model assumptions were evaluated using residual plots and colinearity diagnostics.

To further study structures in the data, PCA was conducted on standardized variables (anti-TPO, iGH, fVAS, FSS, IL−1Ra, IL−6, HSP90α, TNF−α). Principal components and explained variances were examined. Biplots displaying factor loadings for each variable on the two first principal components (PC1 and PC2) were used to visualize joint patterns in PROMs and biomarkers.

A p−value <0.05 was considered statistically significant. Given the exploratory nature of the study, all p-values should be regarded as exploratory and interpreted with caution. Due to the exploratory interpretation no formal adjustment for multiple testing was applied. The analyses were performed in R version 4.4.2.

### Ethical approval and consent to participate

The study received ethical approval from the Regional Committee for Medical Research Ethics in Norway (REK vest 20673). All participants provided written informed consent to participate, and the study was conducted in accordance with the latest revision of the Helsinki Declaration.

## Results

Baseline characteristics including PROMs and biomarkers are given in **Table 1**. The median anti-TPO was 979 IU/mL (IQR 341–2922), reflecting high and heterogeneous antibody titers consistent with active autoimmune thyroid disease.

**Table 1:** Baseline characteristics of 36 patients with autoimmune thyroiditis.

Variable	N=36
Age	49.5 (41-57)
Sex, Female/Male (n, %)	32/4, (89%/11%)
SF-36 GH	16 (10-43)
fVAS	85 (73-97)
FSS	6.7 (5.8-7.0)
IL-1Ra (pg/ml)	532 (351-796)
IL-6 (fg/ml)	2.2 (1.5-3.2)
HSP90α (ng/ml)	20 (19-23)
TNF-α (fg/ml)	322 (245-401)
Anti-TPO (IU/ml)	979 (341-2922)

SF-36 GH, Short Form-36, General Health; fVAS, fatigue Visual Analog Scale; FSS, Fatigue Severity Scale; IL-1Ra, Interleukin-1 Receptor Antagonist; HSP, Heat Shock Protein; TNF, Tumor Necrosis Factor; Anti-TPO, anti-Thyroid peroxidase antibody.

Values are presented as median (Q1–Q3).

### Correlation analysis

Correlation analyses indicated that higher IL−1Ra and IL−6 levels were moderately correlated with poorer general health (higher inverted SF−36 iGH scores), whereas HSP90α and TNF−α showed weaker and non−significant correlations with iGH. IL−6 displayed a positive correlation with fVAS, while HSP90α was inversely correlated with both fVAS and FSS, suggesting that higher HSP90α levels were associated with less fatigue; TNF−α was not significantly related to either fatigue measure (**Table 2**). Anti-TPO showed no significant correlation with any PROM. As expected, the three PROMs were strongly inter−correlated, with the highest correlation observed between iGH and fVAS.

**Table 2:** Correlations between biomarkers and fatigue scales in 36 patients with autoimmune thyroiditis.

Variable	IL-1Ra	IL-6	HSP90α	TNF- α	TPOAb	SF-36 iGH	fVAS	FSS
IL-1Ra		0.001 0.57	n.s 0.26	0.009 0.43	n.s -0.13	0.014 0.41	n.s 0.31	n.s 0.07
IL-6	0.001 0.57		n.s 0.004	0.002 0.50	n.s -0.13	0.037 0.35	0.015 0.40	n.s 0.22
HSP90α	n.s. 0.26	n.s 0.004		n.s 0.19	n.s 0.17	n.s 0.22	0.037 -0.35	0.027 -0.37
TNF- α	0.009 0.43	0.002 0.50	n.s 0.19		n.s 0.09	n.s 0.21	n.s 0.19	n.s -0.05
TPOAb	n.s. -0.13	n.s -0.13	n.s 0.17	n.s 0.09		n.s -0.14	n.s -0.08	n.s -0.28
SF-36 iGH	0.014 0.41	0.037 0.35	n.s 0.22	n.s 0.21	n.s -0.14		0.001 0.68	0.001 0.58
fVAS	n.s 0.31	0.015 0.40	0.036 -0.35	n.s 0.19	n.s -0.08	0.001 0.68		0.002 0.51
FSS	n.s 0.07	n.s 0.22	0.027 -0.37	n.s -0.05	n.s -0.28	0.001 0.58	0.002 0.51	

Spearman’s r (lower triangle) and p-value (upper triangle) are given.

IL-1Ra, Interleukin-1 Receptor Antagonist; IL, Interleukin; HSP, Heat Shock Protein; TNF, Tumor Necrosis Factor; Anti-TPO, Thyroid Peroxidase Antibody; SF-36 iGH, Short Form-36, General Health (inverted); fVAS, fatigue Visual Analog Scale; FSS, Fatigue Severity Scale.

### Regression analysis (univariable)

In age− and sex−adjusted univariable models (**Table 3**), IL−1Ra was significantly associated with poorer general health, explaining approximately 17% of the variance in iGH scores, whereas its associations with fVAS and FSS were not statistically significant. HSP90α demonstrated a borderline inverse association with fVAS. TNF−α and anti-TPO did not show significant associations with any of the PROMs in these univariable models, and age and sex were not significant predictors in any of the separate models.

**Table 3:** Univariable regression in 36 patients with autoimmune thyroiditis.

Variable	SF-36 iGH		fVAS		FSS	
	β	P-value	β	P-value	β	P-value
IL-1Ra	0.03	0.047	0.01	0.23	0.0003	0.56
Age	0.15	0.72	0.17	0.56	0.001	0.93
Sex	12.12	0.37	2.75	0.77	-0.21	0.71
	R^2^=0.169		R^2^=0.066		R^2^=0.013	
IL-6	0.003	0.14	0.001	0.17	0.0001	0.22
Age	0.14	0.75	0.15	0.61	-0.0003	0.99
Sex	16.38	0.24	3.83	0.67	-0.22	0.68
	R^2^=0.121		R^2^=0.079		R^2^=0.048	
HSP90α	-0.04	0.94	-0.74	0.065	-0.04	0.10
Age	0.21	0.63	0.16	0.55	0.0006	0.97
Sex	19.28	0.18	3.70	0.67	-0.23	0.66
	R^2^=0.059		R^2^=0.122		R^2^=0.085	
TNF-α	0.04	0.41	0.04	0.27	-0.0008	0.66
Age	0.09	0.85	0.06	0.84	0.001	0.94
Sex	16.69	0.31	4.38	0.68	0.096	0.88
	R^2^=0.073		R^2^=0.062		R^2^=0.0065	
Anti-TPO	-0.003	0.24	0.00034	0.82	-0.00009	0.31
Age	0.18	0.67	0.20	0.50	0.001	0.94
Sex	13.92	0.34	6.42	0.51	-0.30	0.59
	R^2^=0.100		R^2^=0.024		R^2^=0.035	

Dependent variables are SF-36 iGH, fVAS and FSS in separate models, using selective biomarkers and age and sex as independent variables for each model.

SF-36 iGH, Short Form-36, General Health (inverted); fVAS, fatigue Visual Analog Scale; FSS, Fatigue Severity Scale; IL-1Ra, Interleukin-1 Receptor Antagonist; IL, Interleukin; HSP, Heat Shock Protein; TNF, Tumor Necrosis Factor; Anti-TPO, Thyroid Peroxidase Antibody.

### Multivariable regression

In multivariable models including all four biomarkers together with age and sex, higher IL−1Ra remained independently associated with poorer general health, while anti-TPO, IL−6, HSP90α, TNF−α, age, and sex were not significant predictors of iGH; the full model explained 29% of the variance in iGH scores (**Table 4**). For fVAS, IL−1Ra and HSP90α both showed significant associations in the total model, with higher IL−1Ra relating to more fatigue and higher HSP90α relating to less fatigue, whereas anti-TPO, IL−6, TNF−α, age, and sex were not significant; this model accounted for 43% of the variance in fVAS. For FSS, higher HSP90α was significantly associated with lower fatigue severity, with IL−1Ra showing a positive but non−significant trend, and neither anti-TPO, IL−6, TNF−α, age, nor sex were significantly related to FSS in the full model (R² = 0.270).

**Table 4:** Multivariable regression models for 36 patients with autoimmune thyroiditis.

Variable	SF-36 iGH		fVAS		FSS	
	β	P-value	β	P-value	β	P-value
IL-1Ra	0.04	0.051	0.03	0.014	0.0012	0.10
IL-6	0.001	0.67	0.0008	0.52	0.00006	0.48
HSP90α	-1.11	0.15	-1.70	0.0006	-0.077	0.01
TNF-α	0.03	0.52	0.03	0.32	-0.0007	0.74
Anti-TPO	-0.002	0.22	0.0003	0.82	-0.00006	0.49
Age	-0.07	0.89	-0.13	0.63	-0.009	0.59
Sex	-2.71	0.87	-3.99	0.69	-0.48	0.45
	R^2^=0.287		R^2^=0.427		R^2^=0.270	

Total models including age and sex. Dependent variables are SF-36 iGH, fVAS and FSS applied in separate models.

SF-36 iGH, Short Form-36, General Health (inverted); fVAS, fatigue Visual Analog Scale; FSS, Fatigue Severity Scale; IL-1Ra, Interleukin-1 Receptor Antagonist; IL, Interleukin; HSP, Heat Shock Protein; TNF, Tumor Necrosis Factor; Anti-TPO, Thyroid Peroxidase Antibody.

### Backward selection

IL−1Ra remained the only biomarker retained in the final iGH model, with a β−estimate very similar to the univariable IL−1Ra model, while age and sex remained non−significant; this reduced model explained 16.9% of the iGH variance (**Table 5**). For fVAS, the final model retained both IL−1Ra and HSP90α similarly to the total model; IL-1RA was positively and HSP90α negatively associated with fatigue and explained 36.0% of the variance; IL-6 and TNF−α did not contribute additional explanatory power in the backward−selected model. For FSS, the final model also included IL−1Ra and HSP90α, both showing small but statistically significant opposite associations with fatigue severity, whereas IL-6, TNF−α, age, and sex were excluded; this model explained 19.8% of the variance in FSS scores. Anti-TPO, IL-6, TNF-α, age, and sex were removed by backward selection in all three models. Notably, the set of retained predictors differed by outcome: IL-1Ra was retained in all three models, while HSP90α was additionally retained for fVAS and FSS but not for iGH.

**Table 5:** Multiple regression backwards selection models for 36 patients with autoimmune thyroiditis.

Variable	SF-36 iGH		fVAS		FSS	
	β	P-value	β	P-value	β	P-value
IL-1Ra	0.03	0.047	0.03	0.002	0.001	0.045
HSP90α			-1.56	0.0007	-0.07	0.01
Age	0.15	0.72	0.06	0.81	-0.004	0.82
Sex	12.13	0.38	-7.01	0.40	-0.65	0.23
	R^2^=0.169		R^2^=0.360		R^2^=0.198	

Dependent variables are SF-36 iGH, fVAS and FSS applied in separate models.

SF-36 iGH, Short Form-36, General Health (inverted); fVAS, fatigue Visual Analog Scale; FSS, Fatigue Severity Scale; IL-1Ra, Interleukin-1 Receptor Antagonist; HSP, Heat Shock Protein.

### Principal component analysis

**Figure 1:**
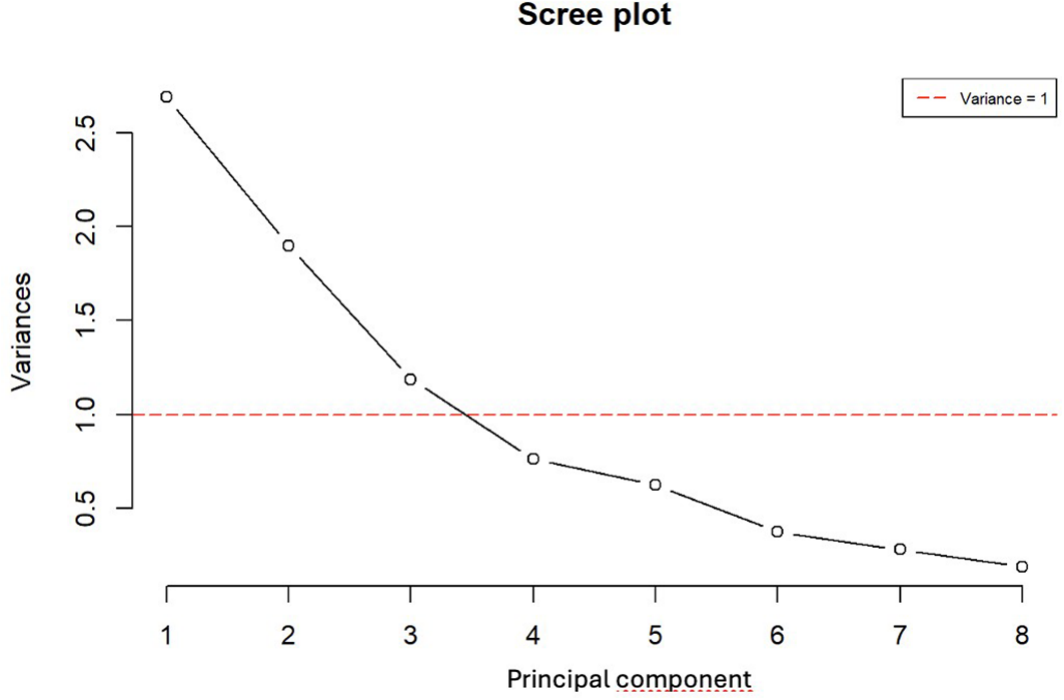
Scree plot illustrates the variance for each principal component of the PCA analysis. Principal components 1, 2 and 3 have variance above 1.0.

**Figure 2:**
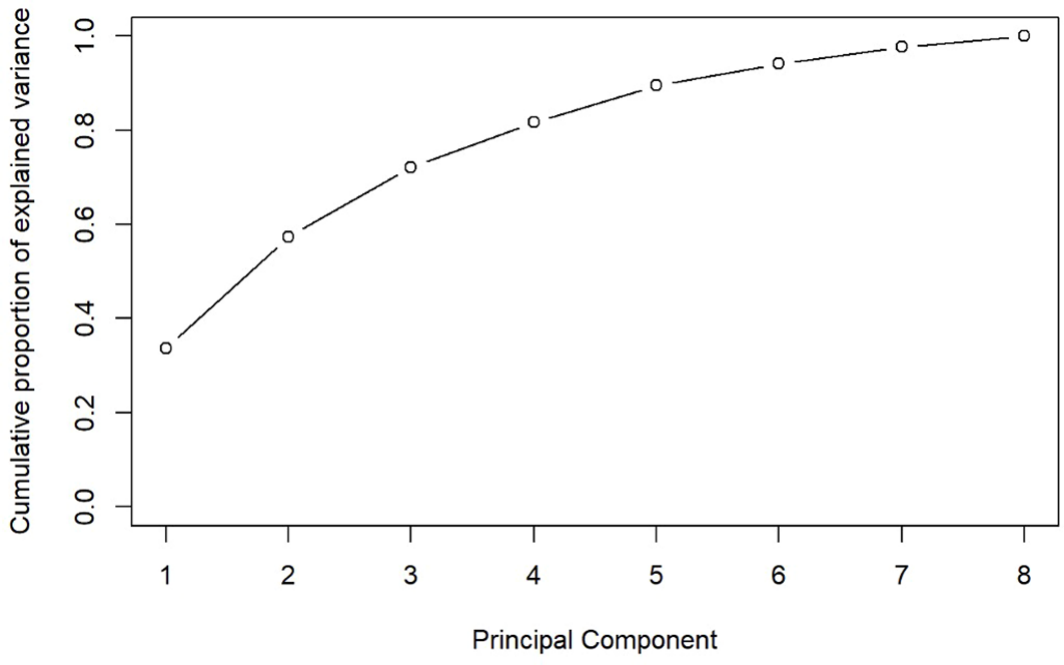
Plot showing the cumulative proportion of explained variance of each principal component of the analysis in 36 patients with autoimmune thyroiditis.

To explore the joint structure of biomarkers and symptom measures, we performed a principal component analysis including anti-TPO, IL−1Ra, IL−6, HSP90α, TNF−α, SF−36 iGH, fVAS, and FSS. The scree plot showed that the first three principal components had variances above 1.0 on standardized variables, showing that together they captured a substantial proportion of the total variance, as also illustrated by the cumulative variance plot (**Figures 1**, **2**).

**Figure 3:**
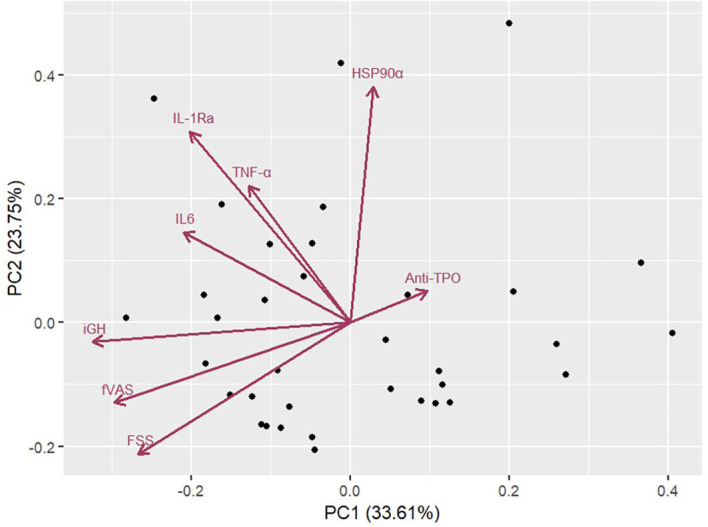
Principal component analysis (PCA) based on selected biomarkers and PROMs at the time of study visit. The arrows show the contributions of each biomarker and PROM to principal component (PC) 1 and 2. iGH, Short Form-36, General Health (inverted); VAS, fatigue Visual Analog Scale; FSS, Fatigue Severity Scale; IL-1RA, Interleukin-1 Receptor Antagonist; HSP, Heat Shock Protein; TNF, Tumor Necrosis Factor; Anti-TPO, Thyroid Peroxidase Antibody.

The PCA biplot indicated that SF−36 iGH, fVAS, and FSS loaded in the same general direction on the first principal component, consistent with a common symptom/fatigue–health dimension (**Figure 3**). IL−1Ra, TNF−α and IL−6 vectors pointed broadly in the same direction as the PROMs, whereas HSP90α loaded opposite to the fatigue measures, in line with its consistent inverse associations with fVAS and FSS in the regression models; TNF−α contributed less distinctly to the first two components. Anti-TPO loaded largely independently of both the PROM cluster and the IL-1Ra/TNF-α/IL-6 cluster, consistent with its absence of significant regression associations with any PROM.

Given the number of variables relative to sample size (8 variables, n=36), PCA results should be regarded as exploratory and potentially unstable across different samples. With this caveat, PC1 could be interpreted as an “inflammatory symptom−burden axis”, where higher PC1 scores correlates with poorer general health, more fatigue, and higher pro−inflammatory biomarker levels. PC2 accounted for an additional proportion of the variance and was driven primarily by HSP90α and IL−1Ra loadings and some contribution from TNF-α, with smaller contributions from PROMs, suggesting a secondary dimension capturing joint variation in IL−1Ra−related activity and HSP90α that is not fully aligned with the main inflammatory symptom−burden axis represented by PC1.

Inspection of PC1–PC2 score plots indicated that individuals with high fatigue and poor general health tended to have high IL−1Ra/IL−6/TNF−α, but showed considerable variation in HSP90α levels, supporting the notion that HSP90α may modify the relationship between inflammation and symptom expression rather than merely mirroring inflammatory load.

## Discussion

The principal finding of this study is that IL−1Ra is the most consistent inflammatory biomarker associated with impaired general health and fatigue in euthyroid patients with Hashimoto’s thyroiditis, with independent associations across correlation, univariable, and multivariable regression models (**Tables 2**–**5**). HSP90α showed an inverse association with fatigue measures in multivariable models and loaded in the opposite direction to PROMs in the PCA, suggesting a potentially protective or modulatory role (**Table 4**; **Figure 3**). IL−6 contributed only in correlation analysis, while anti-TPO and TNF−α did not emerge as an independent determinant of any PROM.

### Symptom burden in euthyroid Hashimoto’s thyroiditis

The cohort displayed a strikingly high symptom burden at baseline, with a median SF−36 GH of only 16 (IQR 10–43), median fVAS of 85 (IQR 73–97), and median FSS of 6.7 (IQR 5.8–7.0), all indicating severe fatigue and markedly reduced general health despite biochemical euthyroidism (**Table 1**). For context, fVAS ≥50 mm is often used as a cut-off for clinically significant fatigue (ref); FSS >4.0 denotes clinically relevant fatigue severity (24); and the population norm for SF-36 GH is approximately 72 (25), underscoring the markedly impaired health status of this cohort. These findings are consistent with the growing body of evidence that a substantial proportion of Hashimoto’s thyroiditis patients experience persistent symptoms despite normalized thyroid hormone levels on levothyroxine treatment or even in euthyroid, untreated individuals with thyroid autoimmunity (8–11). The observation that symptom burden in these patients is largely independent of age and sex in our models (**Tables 3**–**5**) further reinforces the notion that immune−mediated processes, rather than hormonal or demographic factors, are central drivers for symptom generation in this condition.

### IL−1Ra and IL−1 signaling as key correlates of symptom burden

IL−1Ra was the only biomarker significantly associated with poorer general health (iGH) in both the univariable (**Table 3**) and all multivariable models (**Tables 4**, **5**). In the full multivariable fVAS model, IL−1Ra was also independently associated with more fatigue (β = 0.03, p = 0.012), retained significance after backward selection, and contributed to models explaining up to 42.6% of fVAS variance (**Tables 4**, **5**).

IL−1Ra is a naturally occurring competitive antagonist of IL−1 receptor signaling and is produced in excess during active IL−1−driven inflammation as a counter−regulatory mechanism. Circulating IL−1Ra levels therefore serve as a robust surrogate marker for the magnitude of IL−1 pathway activation (26). IL−1 is a key mediator of fatigue across a range of chronic inflammatory and autoimmune conditions, with converging evidence from observational studies and IL−1 blockade trials demonstrating that reduction in IL−1 signaling improves fatigue (17, 18). IL−1β is actively transported across the blood–brain barrier and binds to receptors in fatigue−relevant brain regions, where it modulates neurotransmitter release and activates the “sickness behavior” response - the evolutionarily conserved cerebral program comprising fatigue, anhedonia, and cognitive slowing (27). IL−1Ra, a proxy for IL−1 pathway activity, emerged as the strongest and most consistent biomarker correlate of symptom burden in Hashimoto’s thyroiditis. This extends previous observations to organ−specific autoimmune disease and points to IL−1−mediated neuroimmune signaling as a key mechanism linking thyroid autoimmunity to fatigue and impaired quality of life.

### HSP90α – inverse association with fatigue and possible mechanistic interpretations

A novel finding of this study is the inverse association between plasma HSP90α and fatigue. In the full multivariable model, higher HSP90α was significantly associated with lower fVAS scores and HSP90α loaded opposite to all three PROMs in the PCA biplot (**Table 4**; **Figure 3**). This inverse relationship is intriguing, given that HSP90α is often regarded as a proinflammatory molecule that promotes NF−κB signaling and cytokine production (19).

However, HSP90α has documented context−dependent functions. As a molecular chaperone, it supports cellular “life” and stress defense, and extracellular HSP90α can exert cytoprotective and immunomodulatory effects under certain conditions. In a previous study from our group in patients with Crohn’s disease, higher plasma HSP90α was positively correlated with fatigue severity, and HSP90α was the only significant predictor of fVAS in a multivariable model. The opposite direction of association in the present Hashimoto’s cohort is noteworthy and may reflect differences in the inflammatory milieu, disease chronicity, or the degree of systemic versus organ−confined inflammation between Crohn’s disease and autoimmune thyroiditis. Higher levels of circulating HSP90α may reflect a more effective systemic stress−protective response that buffers fatigue generation. This hypothesis requires validation in larger and longitudinal cohorts.

### Role of IL−6 and TNF−α in Hashimoto−related fatigue

IL−6 showed moderate correlations with poorer general health (iGH) and with fVAS but did not reach significance as an independent predictor in any multivariable model. IL−6 has been linked to fatigue in other conditions, including cancer (14) and rheumatoid arthritis (28), and is elevated in Hashimoto’s thyroiditis compared to healthy controls (29). The modest and model−dependent contribution of IL−6 in our study - present only in correlation analysis and PCA - may reflect both genuine biological overlap with IL−1 pathway signaling and limited statistical power.

TNF−α was correlated with IL−1Ra and IL−6 but showed no significant association with any PROM in univariable or multivariable models (**Tables 2**–**5**). This is somewhat surprising given the established role of TNF−α in sickness behavior and fatigue in other chronic inflammatory populations (30). A possible explanation is that TNF−α exerts its fatigue−related effects indirectly through downstream mediators such as IL−1 and IL−6, and that direct TNF−α–fatigue associations are diluted or masked once these mediators are accounted for. Alternatively, the modest sample size may have reduced power to detect an independent TNF−α–PROM relationship.

### PCA findings: integrated symptom–inflammation axes

PCA provided a complementary, data−driven perspective on the joint structure of biomarkers and symptom measures. PC1 is suggestive of an “*inflammatory symptom−burden axis*”, with iGH, fVAS, FSS, IL−1Ra, and IL−6 all loading in the same direction, consistent with a shared dimension linking proinflammatory IL−1/IL−6 activity to worse patient−reported outcomes (**Figure 3**). PC2 was driven primarily by HSP90α and IL−1Ra loadings, with smaller PROM contributions, indicating a secondary variance dimension in which IL−1Ra–related activity and HSP90α co−vary in a pattern not fully aligned with the main symptom–inflammation axis. This dissociation suggests that HSP90α may modulate how IL-1Ra and IL-6 translates into subjective symptoms, rather than simply reflecting inflammatory load.

### Anti-TPO and clinical symptoms

Anti-TPO was not significantly associated with any PROM in any analytical approach - correlation, univariable regression, multivariable regression, or PCA (**Tables 2**–**5**). This stands in contrast to studies reporting positive associations (5–8). The present findings support the interpretation that anti-TPO is a marker of autoimmune activity rather than a direct driver of symptom generation, and that downstream inflammatory mediators - in particular IL-1 pathway activity - are more proximally linked to fatigue and impaired general health. It should also be noted that, as all participants were anti-TPO positive, the restricted clinical range of anti-TPO levels within this exclusively affected population represents a range restriction issue that may reduce the ability to detect correlations; associations between anti-TPO and symptoms may only emerge across a broader range of values, including anti-TPO-negative controls.

### ​Clinical implications

If confirmed in larger studies, the association between IL−1Ra and fatigue/general health in Hashimoto’s thyroiditis may have several clinical implications. First, IL−1Ra (or other IL−1 pathway markers) could contribute to risk stratification of patients likely to experience persistent fatigue despite biochemical euthyroidism and may be more informative for this purpose than anti-TPO titers, which were not independently associated with symptom burden in the present study. Second, the findings provide a biological rationale for exploring whether interventions targeting IL−1 signaling - already established in other autoinflammatory conditions — could ameliorate fatigue in selected Hashimoto’s patients who do not respond to levothyroxine optimization alone. Third, the divergent behavior of HSP90α relative to proinflammatory cytokines suggests that the relationship between chaperone proteins and symptom generation in autoimmune thyroiditis is more complex than a simple proinflammatory model would predict and warrants further mechanistic investigation. Taken together, these findings suggest that symptom-oriented biomarker assessment in Hashimoto’s thyroiditis should focus on downstream inflammatory mediators of the IL-1 pathway rather than on thyroid autoantibody titers per se.

### Strengths and limitations

Strengths of this study include the well−characterized cohort with confirmed Hashimoto’s thyroiditis (all with anti-TPO >100 IU/mL and ultrasound−verified autoimmune thyroiditis), the use of multiple validated fatigue and HRQOL instruments, the simultaneous measurement of five mechanistically relevant biomarkers including anti-TPO, and the application of complementary statistical approaches (correlation, univariable and multivariable regression, PCA) to provide a comprehensive picture of biomarker–symptom relationships.

​Several important limitations must be acknowledged. First, the sample size of 36 patients limits statistical power, increases the risk of both type I and type II errors, and precludes adjustment for multiple testing. The findings should therefore be considered hypothesis−generating. Second, the cross−sectional design does not permit causal inferences; it remains unknown whether elevated IL−1Ra drives fatigue, reflects a counter−regulatory response to fatigue−generating inflammation, or is merely an epiphenomenon. Third, information on comorbidities - including systemic autoimmune diseases, psychiatric illness, sleep disorders, and other conditions that may independently contribute to fatigue - was not systematically collected, and residual confounding cannot be excluded. Fourth, approximately half the patients were receiving levothyroxine, which may influence both inflammatory markers and symptom expression; however, all patients were euthyroid at baseline, reducing the likelihood that thyroid hormone status confounded the biomarker-PROM associations. A formal subgroup analysis comparing treated versus untreated patients was not feasible given the limited sample size, and future studies should examine whether levothyroxine status modifies the reported associations. Fifth, anti-TPO was measured by routine clinical immunoassay rather than a research-grade platform, which may have introduced greater measurement variability compared to the other biomarkers; this could have attenuated any association between anti-TPO and PROMs. Sixth, with 7 predictors and n=36 patients, the multivariable models including all predictors are at the upper limit of advisable numbers of predictors to include in a multivariable model. No collinearity issues are detected but the model estimates should still be interpreted with caution, as should the selected models after backward selection. Seventh, data on BMI and other potential confounders were not systematically collected, and residual confounding cannot be excluded. Eighth, biomarkers measured in a relevant control group would have strengthened the interpretations.

### Future research

Longitudinal studies with repeated biomarker and PROM assessments - ideally before and after therapeutic interventions such as thyroidectomy or immunomodulatory therapy - are needed to establish the temporal and potentially causal relationships suggested here. Multicenter studies with larger and more diverse cohorts would improve generalizability and allow subgroup analyses, including stratification by levothyroxine use, anti−TPO titer, and comorbidity burden. Mechanistic studies exploring whether IL−1 pathway blockade reduces fatigue in Hashimoto’s thyroiditis, analogous to trials in autoinflammatory syndromes, would provide the most direct test of the hypothesis generated by the present data. Finally, the unexpected inverse association between HSP90α and fatigue in Hashimoto’s thyroiditis, contrasting with positive associations reported in Crohn’s disease, calls for further investigation into the disease−specific and context−dependent roles of extracellular chaperone proteins in fatigue pathophysiology.

## Data Availability

The raw data supporting the conclusions of this article will be made available by the authors, without undue reservation.
